# Could iodine be the missing micronutrient in breast cancer development?

**DOI:** 10.3389/fonc.2026.1674237

**Published:** 2026-04-10

**Authors:** Jia Liu, Xingtong Zhou, Ying Xu, Yidong Zhou, Qiang Sun

**Affiliations:** Department of Breast Surgery, Peking Union Medical College Hospital, Chinese Academy of Medical Sciences & Peking Union Medical College, Beijing, China

**Keywords:** breast cancer, cytoplasmic mislocalization, endocrine dysregulation, iodine intake, Na^+^/I^-^ symporter

## Abstract

Breast cancer is posing a serious threat to the health of the female workforce as one of the most prevalent malignancies. Emerging epidemiological evidence suggests that insufficient intake of the trace element, iodine, is associated with breast oncogenesis. In this paper, we propose a potential link between iodine deficiency and increased breast cancer risk. A central element of this mechanism may involve the cytoplasmic mislocalization and oncogenic function of the sodium/iodide symporter (NIS), which primarily mediates iodine transport under physiological conditions in the human body. We propose a three-stage model: (1) Initiation: Iodine deficiency triggers systemic alterations in the hypothalamic-pituitary-thyroid (HPT) and gonadal (HPG) axes, as well as lactogenic signaling, establishing a proliferative and inflamed breast microenvironment. (2) Heterogeneous subtype-specific evolution: For hormone receptor-positive (HR+) breast cancer, sustained PI3K/AKT activation driven by the relative dominance of estrogen disrupts normal glycosylation, leading to the cytoplasmic retention of NIS. For triple-negative breast cancer, *TP53* and *FOXA1* mutations primarily contribute to significant upregulation of NIS. (3) Malignant evolution: Cytoplasmic NIS associates with the leukemia-associated RhoA guanine exchange factor (LARG) and hyperactivates the RhoA-ROCK signaling pathway, driving cytoskeletal rearrangement, tumor invasion and metastasis. Deciphering the contributions of iodine status toward breast carcinoma development is assumed to open new avenues for novel therapeutic measures for breast cancer.

## Introduction

Iodine (I), a member of the group of halogens, is an essential trace element for human nutrition.

An iodine cycle exists in nature, in which this chemical element is continuously exchanged between the lithosphere, hydrosphere, and atmosphere through biogeochemical processes. The iodine content in water and soil varies in different regions. Thus, the foodstuff from diverse regions features different levels of iodine, which largely determines the iodine nutritional status of local populations. Inhabitants of inland regions (e.g. central Asia and Africa), mountainous areas and flooded areas are highly prone to iodine deficiency (1). Over the past few decades, the promotion of iodized salt has accounted for remarkable progress towards mitigating iodine deficiency disorders. However, iodine insufficiency remains a persistent problem. In 2020, there are still 21 countries lacking adequate iodine in their diet worldwide, partly as a result of local political instability or economic difficulty (2). A report released by the World Health Organization (WHO)/Europe and the Iodine Global Network (IGN) in 2024 indicates that the rising popularity of plant-based alternatives to key sources of iodine, such as dairy products, has led to insufficiency of iodine intake in the WHO European Region and posed an increased health risk (3).

Iodine in food is predominantly absorbed (>90%) in the gut into the bloodstream, and then it is carried to the thyroid and other organs such as stomach and breast via the sodium-iodide symporter (NIS) (2, 4). The thyroid is the primary iodine-concentrating organ, containing 70–80% of the total iodine in the body of a healthy adult. In the thyroid, iodine functions as a crucial component of thyroid hormones thyroxine (T4) and triiodothyronine (T3), which are critical for energy metabolism, thermoregulation and other vital activities. Hypothyroidism occurs in the event of inadequate iodine consumption and further results in a slowing of metabolism throughout the body, triggering systemic harms such as fatigue, obesity, fertility disorders and cognitive decline.

Among the extrathyroidal organs capable of taking up iodine, the breast has gained increasing attention due to the strong correlation between iodine intake and breast health. It has been demonstrated that oral iodine supplementation therapy effectively relieved the symptoms of fibrocystic breast disease and objectively induced regression of fibrosis (5). Under physiological conditions, the expression of the iodide exporter, NIS, is strictly confined to pregnancy and lactation phases in mammary tissue, which are traditionally recognized for their protective role in reducing breast cancer risk. Via NIS activity, the lactating mammary gland efficiently concentrates iodide and thereby supplies breastmilk enriched with iodine for the newborn. This temporally restricted expression pattern suggests a potential link between NIS and mammary gland cell fate.

## Association between iodine status and breast cancer risk

The protective effects of iodine intake on the breast have been proposed. A study by Teas et al. demonstrates that 5% dietary Laminaria supplementation delayed DMBA (7,12-dimethylbenz[a]anthracene)-induced mammary tumorigenesis in rats (6). In 1999, Funahashi and his colleagues came to a similar conclusion that wakame feed suppressed DMBA-induced mammary tumor proliferation in rats without causing thyroid toxicity (7). These results of animal experiments to some extent align with the exceptionally low incidence of breast cancer in Japanese women, who traditionally consume a seaweed-rich diet (8). In contrast, a higher breast cancer incidence was observed in emigration of female Japanese and those adopting westernized nutritional patterns. In addition, Japan, as a country whose exposure to both selenium and iodine is high, demonstrates a lower breast cancer risk than those areas with high selenium but low iodine exposure (e.g., the United States) or regions deficient in both (e.g., Northern Europe) (9). In 2010, a case-control study including 362 cases and 362 controls utilized quantitative food frequency questionnaire (FFQ) to investigate dietary iodine intake and revealed a negative association between gim consumption and breast cancer risk (10). Candidates in the highest quintile of gim intake (median: 2.0 g{dry mass}/day) had a 52% lower risk than those in the lowest intake group (median: 0.06 g{dry mass}/day) (OR = 0.48, 95% CI: 0.27–0.86, *P* = 0.026). Stratified analysis reported a consistent correlation between gim intake and breast cancer in premenopausal women (OR = 0.44, *P* = 0.007) while postmenopausal women exhibited no statistically significant results, indicating combined effects of a complex interplay between iodine intake and estrogen-progesterone levels. By analogy with the above studies, breast carcinoma tissue contains a lower iodine concentration (18.2 ± 4.6 ng I/mg) as compared to benign breast disease tissue (80.9 ± 9.5 ng I/mg) and adjacent normal breast tissue (31.8 ± 4.9 ng I/mg) (11).

Physiologically, more than 90% of absorbed iodine is eliminated through urinary excretion, which renders urinary iodine concentration (UIC) the principal indicator for assessing iodine nutritional status recommended by the WHO. If iodine intake level is associated with breast cancer, this relationship should correspondingly be reflected in UIC levels. Existing clinical observation has provided support for this possible relationship. Eskin et al. demonstrated that women with breast cancer exhibited a statistically lower urinary iodine excretion as compared to non-cancer individuals (105 mcg/L vs. 141 mcg/L, *P* < 0.05) (12). Taken together, these results suggest that sufficient intake of iodine may have the potential to reduce the risk of breast cancer, and that breast cancer is possibly associated with low iodine levels in the body. Here, we propose the hypothesis that iodine deficiency may play a contributory role in the development or progression of breast cancer. While no causal link has been established, this correlation raises the possibility of an underlying biological mechanism that merits further investigation.

## From iodine deficiency to breast cancer: hormonal dysregulation and aberrant NIS cytoplasmic overexpression

At present, the core mechanism by which low iodine intake possibly contributes to breast cancer remains unclear. Interestingly, increased expression of NIS has been observed in human breast cancer across multiple studies. Tazebay et al. first identified that NIS was commonly expressed in human breast cancer samples, with immunohistochemical positivity in over 80% of invasive and ductal carcinoma specimens (13). The prevalent expression of NIS in breast tissue harboring malignancy was subsequently confirmed by larger studies employing diverse molecular techniques for validation (14, 15). More importantly, the transmembrane transport activity of NIS in normal lactating mammary gland is highly dependent on the precise localization at the basolateral membrane of secretory luminal epithelial cells. However, in breast cancer cells, NIS is frequently mislocalized to the cytoplasm (13–15), resulting in a loss of its iodide transport function (16, 17). Furthermore, cytoplasmic accumulation of NIS tissue represents not only loss of normal transport function but also acquisition of an oncogenic signal. The C-terminal region of the NIS protein contains a PDZ-binding motif, enabling physical interaction with the leukemia-associated RhoA guanine exchange factor (LARG). This interaction occurs primarily in the cytoplasm and significantly stabilizes the NIS protein. Lacoste et al. reported that this NIS-LARG complex induced sustained activation of the RhoA signaling pathway, thereby regulating cytoskeletal reorganization and enhancing cancer cell migration and invasion (18). They also found that overexpressing wild-type NIS or the cytoplasmic G543E mutant in HEK293 cells induced epithelial–mesenchymal transition (EMT), marked by downregulated E-cadherin as well as upregulated N-cadherin Vimentin (18). Therefore, the aberrant upregulated expression and cytoplasmic retention of NIS may be a central mechanism for iodine deficiency-related breast cancer pathogenesis.

**Figure 1:**
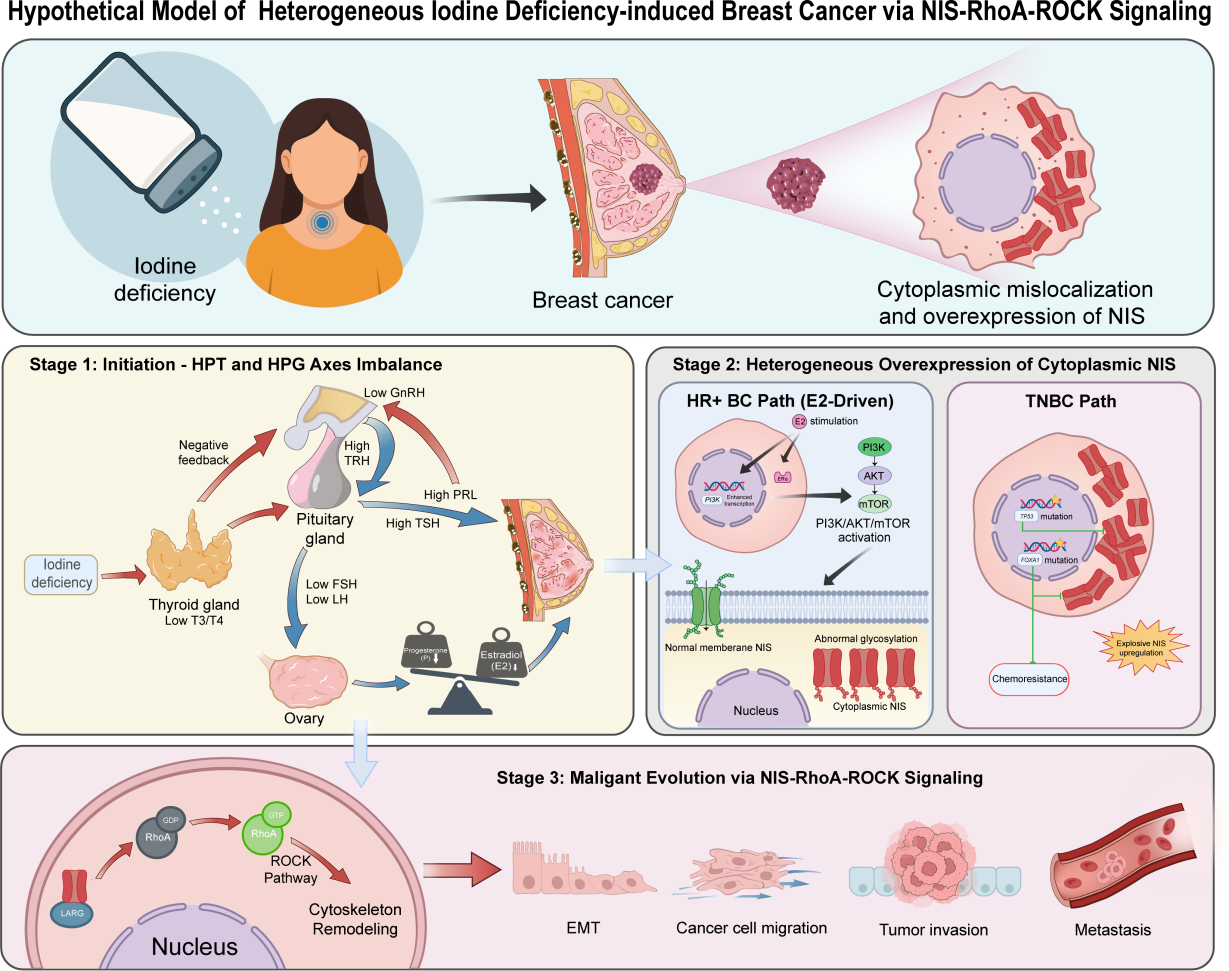
Hypothetical model of heterogeneous iodine deficiency-induced breast cancer via NIS-RhoA-ROCK signaling. Stage 1: Initiation. Iodine deficiency triggers compensatory activation of the HPT axis. The negative feedback due to low T3 and T4 levels causes elevated levels of TRH and TSH, with TRH further inducing high levels of PRL. As a result of low GnRH levels and subsequently low FSH and LH levels, progesterone (P) levels decline significantly more than estradiol (E2) levels in the presence of luteal dysfunction. Therefore, an imbalance in the E2/P ratio occurs and establishes a highly proliferative and inflammatory breast microenvironment. Stage 2: Heterogeneous Overexpression of Cytoplasmic NIS. In the HR+ BC path, continuous E2 stimulation induces PI3K/AKT/mTOR pathway activation by ER-mediated transcriptional regulation of PI3K-related genes and direct interaction between estrogen receptor α (ERα) and PI3K subunits, leading to abnormal NIS glycosylation and its subsequent cytoplasmic translocation. In the TNBC path, *TP53* and *FOXA1* mutations trigger explosive NIS upregulation. Stage 3: Malignant Evolution. Cytoplasmic NIS loses its iodine-transport function and associates with LARG to activate the RhoA-ROCK signaling pathway, acting as an oncogenic scaffold. Consequently, this pathway activation drives cytoskeletal rearrangement, EMT, tumor invasion, and metastasis. Green NIS, Normal membrane-bound NIS; Red NIS, Abnormal cytoplasmic NIS. T3, triiodothyronine; T4, thyroxine; TRH, thyrotropin-releasing hormone; TSH, thyroid-stimulating hormone; PRL, prolactin; GnRH, gonadotropin-releasing hormone; FSH, follicle-stimulating hormone; LH, luteinizing hormone; E2, estradiol; ERα: Estrogen receptor alpha; HR+ BC, hormone receptor-positive breast cancer; TNBC, triple-negative breast cancer; LARG, Leukemia-associated Rho guanine nucleotide exchange factor; EMT, epithelial-mesenchymal transition.

In this study, we sought to bridge the gap between iodine deficiency and breast cancer evolution via aberrant NIS overexpression in the cytoplasm as an oncogenic scaffold. **Figure 1** outlined a hypothetical model and potential mechanism.

### Stage 1: initiation - HPT and HPG axes imbalance

It is well known that iodine deficiency impairs thyroid hormone production and serves as a major cause of hypothyroidism (19), directly disrupting the hypothalamus-pituitary-thyroid (HPT) axis and further impacting the hypothalamic-pituitary-gonadal (HPG) axis. Low thyroid hormone levels caused by iodine deficiency trigger negative feedback on the hypothalamus and pituitary gland, giving rise to elevated thyrotropin-releasing hormone (TRH) and thyroid-stimulating hormone (TSH) levels. Consequently, prolactin (PRL) levels are elevated due to the stimulation of pituitary lactotrophs by increased TRH. Under physiological conditions, NIS in the mammary gland is positively regulated by PRL levels (20, 21). PRL has been also found to dose-dependently increase NIS mRNA levels in 3D histocultured human breast tumors (22). Further to this point, Rillema et al. demonstrated that PRL treatment upregulated NIS protein expression in mouse mammary gland explants via laser densitometry of Western blots (23). These results strongly suggest that NIS expression could be possibly upregulated by increased PRL in the event of iodine deficiency. On the other hand, a previous study by Saito et al. has also demonstrated that TSH treatment upregulated NIS mRNA and protein expression through the cAMP signaling in thyroid follicular cells (24), though its role in mammary NIS regulation remains controversial (25). Meanwhile, enhanced PRL production can inhibit the secretion of gonadotropin-releasing hormone (GnRH) in the hypothalamus, leading to reduced secretion of follicle-stimulating hormone (FSH) and luteinizing hormone (LH) (26). This interference manifests as ovulatory dysfunction, luteal insufficiency, and therefore a marked decline in progesterone (P) levels in female. Under these circumstances, a “relative estradiol (E2) dominance” could be established (27). Long-term high estradiol stimulations may promote inflammatory responses via producing quinone compounds during its metabolism and generating reactive oxygen species (ROS) as well as enhancing the activity of the transcription factor NF-κB in the localized microenvironment (28–30). Thus, iodine deficiency-induced endocrine dysregulation involving HPT and HPG axes imbalance may upregulate NIS expression and create an inflammatory environment in the mammary gland, potentially increasing cancer risk.

### Stage 2: heterogeneous overexpression of cytoplasmic NIS

The aberrant expression of NIS has been documented in different molecular subtypes of breast cancers (31, 32), whereas the inherent mechanism may vary across diverse subtypes. In hormone receptor-positive (HR+) breast cancer, the PI3K/AKT/mTOR signaling pathway is prevalently activated (33). Elevated estradiol levels can further enhance its activation via nuclear estrogen receptor (ER) genomic and non-genomic mechanisms (34). Knostman et al. demonstrated that upregulated expression of NIS was induced by PI3K signaling in transgenic mouse models of breast cancer (35). In addition, NIS must undergo complete N-glycosylation (about 65–85 kDa) for proper trafficking to the cell membrane under normal conditions (36). However, in PI3K/AKT-activated MCF-7 human mammary carcinoma cells, NIS existed as a 50 kDa underglycosylated form lacking membrane localization signals, leading to its retention in the cytoplasm (37). This association raises the possibility that persistent relatively predominant estradiol induces the activation of the PI3K pathway, leading to upregulated expression and abnormal glycosylation of NIS in HR+ breast cancer.

In contrast, the mechanistic framework explaining the aberrant NIS expression is less comprehensively described for triple-negative breast cancer (TNBC) in the existing literature. TNBC is recognized to be primarily associated with mutations in the *TP53* gene. Evidence suggests that p53 suppresses NIS expression in multiple breast cancer cell types and mice model (38). Frequent *TP53* mutations could potentially lead to significant upregulation of NIS in TNBC (32). Additionally, Demyashkin et al. identified a close association between NIS expression in TNBC and the activity of the transcription factor *FOXA1* whose expression and methylation vary depending on a woman’s gravidity status (39). They proposed that higher NIS expression could possibly correlate with worse prognosis and resistance to neoadjuvant chemotherapy as a result of *FOXA1* activity. Based on this, a speculative driving mechanism of abnormal NIS expression in TNBC may involve *TP53* and *FOXA1* mutations.

### Stage 3: malignant evolution via RhoA-ROCK signaling

As noted earlier, the accumulated NIS in the cytoplasm associates with LARG, forms NIS-LARG complex, and then activates RhoA. Upon stimulation, RhoA undergoes a conformational change by releasing GDP and binding GTP, thereby triggering downstream ROCK signaling cascades to facilitate stress fiber formation, EMT, cell migration and invasive behavior.

## Hypothesis

In this paper, we propose that sufficient iodine intake protects the breast from oncogenesis while inadequate iodine consumption is a risk factor for breast cancer. In this specific causal chain, we hypothesize that iodine deficiency-induced systemic endocrine disruption including HPT and HPG axes and PRL signals, drives breast cancer malignant progression by triggering the cytoplasmic internalization of NIS, which acts as an oncogenic scaffold to activate the RhoA-ROCK signaling pathway.

### Preliminary evaluation of the hypothesis

**Figure 2:**
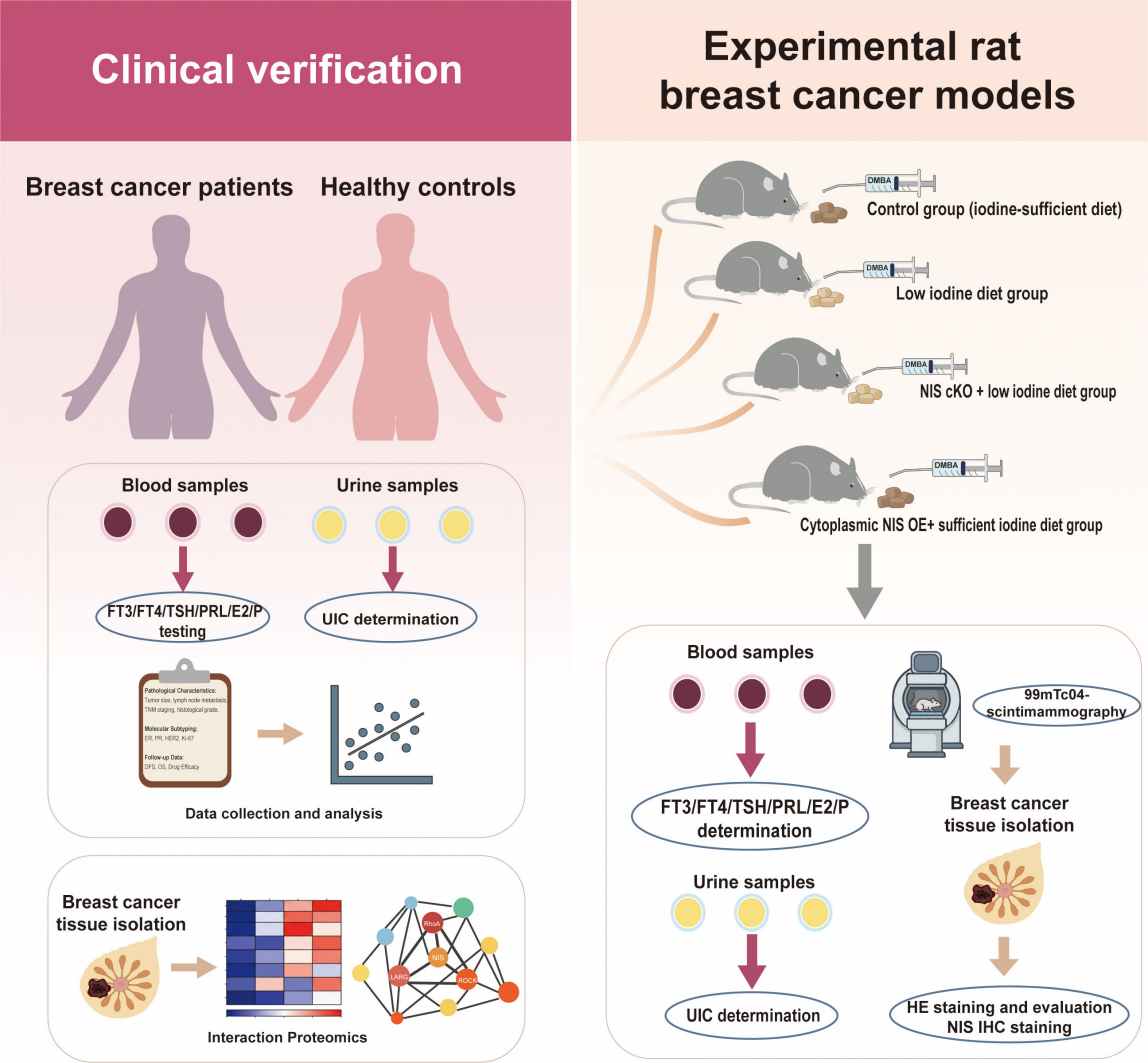
Overview of the preliminary experimental design. FT3, free triiodothyronine; FT4, free thyroxine; TSH, thyroid-stimulating hormone; PRL, prolactin; E2, Estradiol; P, progesterone; DMBA, 7,12-dimethylbenz[a]anthracene; cKO, conditional knockout; OE, Overexpression; 99mTcO4-, technetium-99m pertechnetate.

In order to preliminarily test our hypothesis, we will conduct both clinical and basic experimental studies (**Figure 2**). First of all, the associations between UIC and breast cancer will be initially evaluated through a cross-sectional study. Urine samples from newly diagnosed, untreated breast cancer patients and healthy control subjects will be collected to compare their UIC levels. Further subgroup analyses will be conducted within the breast cancer cohort to explore the association between UIC and specific clinicopathological characteristics such as tumor size, multifocality and axillary lymph node metastasis status. To investigate whether abnormal iodine status serves as an independent risk factor for axillary lymph node metastasis, binary logistic regression analysis will be performed. FT3, FT4, TSH, PRL, E2, and P status in the blood of breast cancer patients and healthy individuals will also be assessed to determine whether the results align with the hormone imbalance pattern suggested by our hypothesis. Follow-up data including overall survival (OS) and disease-free survival (DFS) will be prospectively collected for use in future research. To elucidate the oncogenic function of NIS, we plan to perform co-immunoprecipitation coupled with mass spectrometry (Co-IP MS) on the cytosolic fractions of breast cancer tissues derived from samples of surgical patients. This interactomics approach will map the cytoplasmic NIS protein-protein interactions network, aiming to confirm its physical association with key cytoskeletal regulators such as LARG, RhoA, and ROCK.

To verify whether different iodine statuses influence breast carcinogenesis and the specific role of NIS expression, a chemically DMBA induced rat breast cancer model could be employed (40). The experimental animals will be randomly assigned into the following four groups: control group (receiving an iodine-sufficient diet), low iodine diet (LID) group, NIS cKO + LID group (NIS conditional knockout in the breast combined with low iodine feed) and cytoplasmic NIS overexpression + sufficient iodine diet group. *Slc5a5*^fl/fl^ mice will be crossed with *MMTV-Cre* transgenic mice to induce targeted deletion of the *NIS* gene in the breast tissue. To induce specific cytoplasmic NIS overexpression, a glycosylation-deficient NIS mutant will be generated by substituting asparagine with glutamine at positions 225, 485, and 497 via site-directed mutagenesis (41). This mutant construct will be then delivered into the mammary epithelium of rats through intraductal injection. The sample size for each group of rats will be set at 15. Custom-formulated purified feed will be adopted for all experimental diets. The LID group and NIS cKO + LID group should be given deionized distilled water. Selenium content (0.15 mg/kg according to National Research Council) and other micronutrients should be matched for to minimize the influence of confounding factors. Prior to DMBA administration, the rats will be maintained on different diet patterns for 1 month. Subsequently, DMBA (20 mg/kg) will be administered via gastric gavage as an inducer for breast cancer. After administration, the original dietary regimen will be maintained with the experiment scheduled to conclude around week 20. Meanwhile, the important hormones in our hypothesis regarding the HPT and HPG axes of the rats (FT3, FT4, TSH, PRL, E and P) as well as UIC will be also monitored every four weeks during the experiment. The severity of iodine deficiency will be determined mainly by TSH and UIC levels. After stable tumor formation, the functional iodine uptake in rat mammary tissues of each group will be measured by technetium-99m pertechnetate (99mTcO4^-^) scintimammography using a small-animal Micro-SPECT/CT system. Then the rats will be euthanized and their mammary tissue will be harvested for HE staining to evaluate tumor multiplicity and size. NIS expression level and its location in rat mammary glands of the four groups will be examined by immunohistochemical (IHC) staining. The outcomes of this experiment might shed light on the mechanism by which iodine nutritional status affects breast cancer risk.

### Statistical analysis

Notably, UIC will be expressed as median and interquartile range (IQR) because of their skewed distribution. To compare UIC levels between the healthy control group and the breast cancer cohort, the Mann-Whitney U test will be employed. Within the breast cancer cohort, differences in UIC across various clinicopathological subgroups (e.g., tumor size, multifocality, and axillary lymph node status) will be analyzed using the Kruskal-Wallis H test or the Mann-Whitney U test, as appropriate. Categorical variables will be compared using the Chi-square test or Fisher’s exact test. To investigate whether abnormal iodine status serves as an independent risk factor for axillary lymph node metastasis, binary logistic regression analysis will be performed. UIC of breast cancer patients will be categorized into three groups according to the WHO guidelines (Deficiency: UIC ≤ 99μg/L, Adequate: 100–199 μg/L, Above requirements: ≥ 200 μg/L) and the WHO-defined “Adequate” group will be set as the reference category. Both univariate and multivariate logistic regression models will be constructed to calculate the odds ratios (ORs) and 95% confidence intervals (CIs). The multivariate model will adjust for potential confounding variables such as age, BMI, and tumor size. All statistical tests will be two-sided, and a *P*-value< 0.05 will be considered statistically significant.

The sample size calculation process for animal experimental models is shown below: Tumor multiplicity was set as the primary endpoint with a minimum mean difference of 3 (assuming a standard deviation of 2.5) with 80% power and a two-sided α of 0.05 (42, 43). Considering the anticipated 20% animal attrition rate in long-term experiments and referencing previous relevant studies, we calculated a sample size of 15 using the formula and values below (44, 45).


$$n=\frac{2\sigma^{2{\left(Z_{\frac{\alpha }{2}}+Z_{\beta }\right)}^{2}}}{d^{2}}$$

n= Required sample size per group.

σ= Expected standard deviation (2.5).

$Z_{\alpha /2}$= Critical value for a two-sided alpha of 0.05 (1.96).

$Z_{\beta }$= Critical value for a power of 80% (0.84).

d= Expected clinically meaningful difference between the two group means (3).

## Discussion

Current research concerning the relationship between iodine intake and breast cancer development has several limitations that warrant consideration. Firstly, there is a lack of prospective clinical cohorts and randomized controlled trials to definitively confirm the relationship between dietary iodine intake and breast cancer risk, which is understandable to some degree given the substantial costs required for long-term follow-ups. Secondly, existing clinical studies have primarily focused on the correlation between iodine intake and breast cancer incidence. However, the assessment methods adopted are not uniform as they include FFQ, diet history interview, and UIC determined using the ammonium persulfate method or ion exchange chromatography (12, 46). The FFQ method relies on subject recall, inevitably suffering from recall bias, and is meanwhile affected by regional variations in iodine content of food. Thirdly, few studies have investigated changes in iodine levels among breast cancer patients and whether these variations correlate with distinct clinicopathological characteristics or prognostic outcomes. In parallel, the related basic research has been somewhat fragmented. While some studies have demonstrated the impact of iodine intake on breast tumorigenesis, others have independently identified the aberrant subcellular localization of NIS in breast cancer cells. Yet, these findings are rarely integrated. The current literature emphasizes localized effects within the breast cancer cells without addressing the relationship among clinical outcomes, endocrine dysregulation, and molecular alterations. Therefore, our hypothesis seeks to bridge this critical gap by integrating these fragmented evidences into a mechanistic framework involving endocrine imbalance and microscopic cellular transformation to explain the role of NIS in breast cancer. As for the validation plan, we aim to preliminarily investigate the impact of iodine status on both breast cancer susceptibility and clinicopathological characteristics utilizing UIC as a reliable metric in our own cohort. Through the application of interactomics and rat models, we seek to systematically validate the mediating role of NIS in this process, thereby offering a more holistic perspective on the iodine-breast cancer axis.

Based on our hypothesis, we suggest that maintaining adequate iodine levels through dietary sources may play a protective role against breast cancer. Importantly, iodine intake exhibits a clear U-shaped relationship with the risk of thyroid diseases such as thyroid nodules and hypothyroidism, meaning that both iodine deficiency and excess significantly increase morbidity (47). In regions with excessive iodine intake (median urinary iodine ≥ 300 μg/L), the cumulative incidence of subclinical hypothyroidism was reported at 2.9% in contrast to 0.2% in mildly deficient cohorts. Furthermore, higher iodine levels were positively correlated with a rise in autoimmune thyroiditis, with incidence rates increasing from 0.2% in deficient areas to 1.3% in iodine-excessive regions (48). The tolerable upper intake level (UL) for adults is 1,100 μg/day according to National Academy of Medicine (NAM). A randomized, controlled clinical trial in Wales also showed that total iodide intakes of 750 μg/day or more significantly elevated TSH concentrations (49). The favorable iodine intake levels mentioned in our study such as gim (2.0 g/day) and UIC (141 mcg/L) are unlikely to trigger the adverse thyroid effects associated with high-dose supplementation. Nonetheless, it is crucial to regularly monitor iodine intake levels and TSH levels when attempting to use an iodine-rich diet for breast health.

Could the relationship between iodine intake and breast cancer risk also follow a U-shaped pattern? Epidemiological and experimental data regarding iodine status and breast cancer risk appears to suggest that excessively high iodine levels may also undermine breast health. A case-control study in Turkey involving 24 breast cancer patients and 48 controls showed a significantly higher proportion of high UIC levels (>200 μg/L) among breast cancer patients (46). He et al. reported that urinary iodine levels markedly increased on the first postoperative day in premenopausal breast cancer patients (a mean ratio of 1012.5 ± 752.2 μg/g creatinine), likely due to transdermal absorption of povidone-iodine (PVP-I) applied during surgery (50). This rapid increase far exceeds not only the urinary iodine levels achievable through daily diet but also the excessive iodine intake threshold defined by the WHO. He and his colleagues further simulated excessive iodine exposure by rubbing iodine tincture onto the backs of nude mice, discovering that this overdosed iodine treatment promoted tumor cell proliferation by stimulating the transcriptional activity of estrogen receptor α. Excess iodine also inhibits thyroid hormone synthesis, leading to the Wolff-Chaikoff effect, which can also cause hypothyroidism featuring an upregulated TSH level. In this context, a hormonal imbalance partially resembling our hypothesis might arise, probably increasing the risk of breast cancer. Furthermore, at supraphysiological concentrations, iodine can induce excessive ROS production and lipid peroxidation. Therefore, we hypothesize that the optimal anti-cancer benefit of iodine lies within a specific therapeutic window, and that exposure to excessive iodine could theoretically exert adverse effects on the breast, highlighting the need for precise dosage evaluation in future applications.

Therapeutic strategies targeting iodine uptake pathways still warrant further exploration. An iodine-rich diet in daily life might represent a possible avenue for breast cancer prevention and management. It is noteworthy that pregnant women are more susceptible to iodine deficiency and hypothyroidism because of the increased need for iodine during pregnancy (51). Women during pregnancy and lactation are recommended to be more intensively supplemented with iodine (250 µg recommended daily allowance, RDA), while the RDA for adults is 150 µg. The actual iodine intake of pregnant and lactating women commonly fails to meet the recommended amount (52). Public health policies should strengthen iodine nutritional surveillance and intervention. Furthermore, different means of iodine-related therapy should be explored in order to assist breast cancer treatment. Stoddard et al. demonstrated that iodine/iodide treatment suppressed the mRNA levels of the estrogen responsive genes *TFF1* and *WISP2* (53), indicating the potential for chemotherapeutic sensitization. Special drug treatment may increase the expression of normal iodide-uptaking NIS in breast cancer, thereby enabling radioiodine therapy to take effect. Kogai et al. reported that all-trans retinoic acid (tRA) significantly upregulated fully glycosylated NIS expression in postnuclear membrane fractions and promotes radioactive iodine (¹³¹I) uptake and retention in ER+ MCF-7 breast cancer cells selectively (54). These results lend some support to the cytoplasmic overexpression of NIS caused by incomplete glycosylation in HR+ breast cancer in our hypothesis. Meanwhile, they represent possible alternative strategies for specific breast cancer subtypes in case of drug resistance. In addition to iodide which serves as the primary circulating form of iodine in the body, breast tissue also harbors trace molecular iodine (I_2_) through direct exogenous intake and local enzymatic iodide oxidation rather than NIS-mediated transport system. I_2_ reacts with arachidonic acid in breast tissue to form 6-iodolactone (6-IL), which may exert anti-proliferative and pro-apoptotic effects on tumor cells by activating peroxisome proliferator-activated receptor gamma (PPAR-γ). Oral supplement of I_2_ has been reported to facilitate the neoadjuvant therapy 5-fluorouracil/epirubicin/cyclophosphamide or Taxotere/epirubicin in breast cancer patients, with I_2_-treated tumors exhibiting lower invasiveness and enhanced antitumoral immune response (55, 56).

Finally, we must acknowledge the boundary between correlation and causation in our study. In fact, our hypothesis originated from the results of clinical observations and basic science studies. By integrating the potential mechanisms of these findings, we have synthesized the observed associations and provided a theoretical rationale for iodine intake and breast cancer risk rather than an established causal relationship. The epidemiological studies regarding iodine status and breast cancer are mainly cross-sectional, only representing a strong association but cannot rule out confounding factors. Similarly, while the cell and animal experiments demonstrate biological plausibility regarding the influence of PRL and estradiol on NIS Expression and the effect of NIS cytoplasmic overexpression on breast carcinogenesis, they do not perfectly mimic human breast cancer pathogenesis. In reality, this process from iodine deficiency to breast cancer cannot be viewed in isolation and is inevitably influenced by confounding factors currently unaccounted for in our hypothesis and preliminary validation plan. Environmental exposures, particularly endocrine-disrupting chemicals (EDCs), could possibly aggravate the breast cancer risk associated with inadequate iodine intake. Contaminants such as perchlorate, thiocyanate, and nitrate can inhibit NIS-mediated iodide uptake and therefore may cause relative iodine deficiency even with adequate dietary iodine intake, eventually triggering the “Initiation” stage proposed in our hypothesis (57). Besides, a recent Mendelian randomization study demonstrated that specific EDCs exhibiting estrogen-like activity such as Bisphenol F (BPF), can alter circulating metabolites and significantly increase the risk of ER+ breast cancer (58). Thus, exposure to estrogen-mimicking EDCs could exacerbate a hyper-estrogenic environment, thereby accelerating the pathological evolution toward NIS mislocalization in HR+ breast cancer subtypes. Furthermore, dietary factors beyond iodine intake—such as the consumption of phytoestrogens, high-fat diets, or alcohol—have been identified to modulate estrogen receptor signaling and systemic inflammation (59–61). Genetic predisposition may also act synergistically with iodine status. While our hypothesis model involves *TP53* and *FOXA1* alterations for TNBC, an individual’s inherent genetic susceptibility to these mutations will likely dictate their threshold for NIS oncogenic transformation. In summary, while iodine deficiency provides a compelling trigger for breast cancer development and evolution, our hypothesis requires subsequent validation. Future investigation into the underlying mechanism should incorporate multifactorial models to better clarify this association and reveal the exact role of iodine deficiency.

## Data Availability

The original contributions presented in the study are included in the article/supplementary material. Further inquiries can be directed to the corresponding authors.
